# Draft Genome Sequences of *Achromobacter piechaudii* GCS2, *Agrobacterium* sp. Strain SUL3, *Microbacterium* sp. Strain GCS4, *Shinella* sp. Strain GWS1, and *Shinella* sp. Strain SUS2 Isolated from Consortium with the Hydrocarbon-Producing Alga *Botryococcus braunii*

**DOI:** 10.1128/genomeA.01527-15

**Published:** 2016-01-14

**Authors:** Katy J. Jones, Karen Moore, Christine Sambles, John Love, David J. Studholme, Stephen J. Aves

**Affiliations:** Department of Biosciences, College of Life and Environmental Sciences, University of Exeter, Exeter, United Kingdom

## Abstract

A variety of bacteria associate with the hydrocarbon-producing microalga *Botryococcus braunii*, some of which may influence its growth. We report here the genome sequences for *Achromobacter piechaudii* GCS2, *Agrobacterium* sp. strain SUL3, *Microbacterium* sp. strain GCS4, and *Shinella* sp. strains GWS1 and SUS2, isolated from a laboratory culture of *B. braunii*, race B, strain Guadeloupe.

## GENOME ANNOUNCEMENT

*Botryococcus braunii* is a green microalga that is able to produce and accumulate high levels of hydrocarbons (up to 60% of its dry weight, depending on the strain), which can be converted into liquid fuels (1, 2). Strains of *B. braunii* are typically not axenic but consist of a single algal strain accompanied by a variety of microorganisms associated as both a biofilm and planktonic population in the water column, which may greatly influence *B. braunii* growth and hydrocarbon production (3–6).

Five bacterial strains (GCS2, SUL3, GCS4, GWS1, and SUS2) were isolated from a long-term laboratory culture of *B. braunii* strain Guadeloupe (race B) (7), and the colonies were grown on LB plates. Genomic DNA fragmented by sonication was concentrated and purified using a QIAquick column (Qiagen), and genomic libraries were prepared using the NEBNext DNA Library Prep Master Mix Set for Illumina (New England BioLabs). One hundred fifty-base pair paired-end sequencing, with a custom bar code, was carried out on an Illumina MiSeq, and *de novo* assembly of the sequence data was carried out using SPAdes version 3.5.0 (8). The number of contigs, coverage, *N*_50_, and total sequence length were, respectively, 20, 41×, 626,645, and 6,180,134 bp for *Achromobacter piechaudii* GCS2; 72, 52×, 358,737, and 6,013,863 bp for *Agrobacterium* sp. strain SUL3; 7, 73×, 944,354, and 3,652,908 bp for *Microbacterium* sp. strain GCS4; 141, 44×, 176,944, and 6,989,725 bp for *Shinella* sp. strain GWS1; and 112, 32×, 264,748, and 7,005,767 bp for *Shinella* sp. strain SUS2. The genomes were annotated using the NCBI Prokaryotic Genome Annotation Pipeline (PGAP), which predicted 5,433, 5,585, 3,325, 6,636, and 6,361 coding sequences (CDSs), respectively, for the five strains. The G+C contents varied between 59.2% (*Agrobacterium* sp. SUL3) and 69.5% (*Microbacterium* sp. GCS4). The 16S rRNA gene sequences were extracted using RNAmmer (9).

Phylogenetic analysis of 16S rRNA gene sequence, *atpD*, *recA*, *rpoB*, and *tyrB* suggested that GCS2 belongs to the species *A. piechaudii*; its genome shares 98.4% average nucleotide identity (ANI) with that of *A. piechaudii* strain ATCC 43553. Phylogenetic analysis of 16S rRNA gene sequence, *gyrB, ppk*, *recA*, and *rpoB* suggested that GCS4 belongs to the genus *Microbacterium*. Phylogenetic analysis of 16S rRNA gene sequence, *atpD*, and *recA* suggested that GWS1 and SUS2 both belong to the genus *Shinella*, and SUL3 belongs to the genus *Agrobacterium*.

None of these five bacteria were previously reported in association with *B. braunii*, except for *Shinella* sp. GWS1 and SUS2, which, on the basis of partial 16S rRNA gene sequence, are indistinguishable from a biofilm isolate found to enhance the growth of a laboratory-maintained monoculture of *B. braunii* (4) (reported as *Rhizobium* sp. M14). Many species of algae are auxotrophic for vitamin B_1_ (thiamine), vitamin B_7_ (biotin), and vitamin B_12_ (cobalamin) (10). We identified genes encoding pathways for thiamine synthesis in all five genomes; cobalamin is likely to be synthesized by *Agrobacterium* sp. SUL3 and *Shinella* spp. GWS1 and SUS2, while biotin is likely to be synthesized by *A. piechaudii* GCS2 and *Agrobacterium* sp. SUL3. No evidence of nitrogen fixation genes was found in any of the bacterial genomes, based on tBLASTn and BLASTn searches.

### Nucleotide sequence accession numbers.

These genome sequences have been deposited at GenBank under the following accession numbers: *A. piechaudii* GCS2, LGYD00000000; *Agrobacterium* sp. SUL3, LGZB00000000; *Microbacterium* sp. GCS4, LGYE00000000; *Shinella* sp. GWS1, LGYF00000000; and *Shinella* sp. SUS2, LGYG00000000. The versions described in this paper are the first versions: LGYD01000000, LGZB01000000, LGYE01000000, LGYF01000000, and LGYG01000000.
